# From Macronutrients to Functional Foods: Nutritional Strategies for Recovery Following High‐Intensity Exercise

**DOI:** 10.1002/fsn3.72331

**Published:** 2026-09-06

**Authors:** Haydar Rafsya Farzana, Gosy Endra Vigriawan, Antonello Santini, Fahrul Nurkolis

**Affiliations:** ^1^ Faculty of Sports Science Universitas Negeri Malang Malang East Java Indonesia; ^2^ Department of Pharmacy University of Napoli Federico II Napoli Italy; ^3^ Faculty of Medicine Universitas Airlangga Surabaya Indonesia; ^4^ Pharmaceuticals, Nutrition, Biomedical Sciences, and Translational Medicine Medical Research Center of Indonesia Surabaya Indonesia; ^5^ Institute of Research and Community Service State Islamic University of Sunan Kalijaga Yogyakarta Indonesia; ^6^ Fahrul Institute for Innovation and Research (FIIR) Yogyakarta Indonesia

**Keywords:** exercise‐induced muscle damage, muscle soreness, nutritional strategies, polyphenols, postexercise recovery, sports nutrition

## Abstract

High‐intensity exercise improves aerobic and anaerobic performance, neuromuscular function, and sport‐specific readiness but may also induce glycogen depletion, exercise‐induced muscle damage, muscle soreness, inflammatory responses, oxidative stress, and temporary reductions in subsequent performance. Recovery nutrition is therefore essential to restore physiological readiness. This review aimed to synthesize current evidence on nutritional strategies for recovery following high‐intensity exercise, ranging from foundational macronutrients to functional foods and bioactive compounds. A structured narrative review was conducted using peer‐reviewed literature retrieved from PubMed/MEDLINE, Scopus, Web of Science, SPORTDiscus, ScienceDirect, and Google Scholar. Priority was given to position stands, consensus statements, systematic reviews, meta‐analyses, randomized controlled trials, and human intervention studies published in English between 2011 and May 2026. Eligible studies involved athletes or physically active adults and examined biochemical, perceptual, or functional recovery outcomes after high‐intensity, repeated‐sprint, resistance, eccentric, or muscle‐damaging exercise. Carbohydrate and protein remain the most consistently supported nutritional strategies for recovery. Carbohydrate facilitates glycogen resynthesis, particularly when recovery time is limited, whereas high‐quality protein supports muscle protein synthesis and tissue repair. Functional foods and bioactive compounds may provide additional benefits as adjunct recovery strategies, although the available evidence varies considerably across exercise models, participant characteristics, supplementation protocols, and recovery outcomes. Among the functional foods reviewed, tart cherry currently has the strongest supporting evidence for selected recovery outcomes. Curcumin, omega‐3 fatty acids, and beetroot have shown promising context‐dependent benefits, whereas evidence for pomegranate, cocoa flavanols, and green tea remains more variable and is generally stronger for oxidative‐stress modulation than for consistent improvements in functional recovery. Chronic high‐dose vitamin C and E supplementation may impair training adaptations. Based on the synthesized evidence, we propose a hierarchical and periodized conceptual framework for recovery nutrition following high‐intensity exercise, in which carbohydrate and protein adequacy remain the primary priorities. Within this framework, functional foods and bioactive compounds may serve as context‐dependent adjuncts, and their application should be individualized according to recovery demands, competition schedule, adaptation goals, and the current strength and consistency of the available evidence.

## Graphical abstract

Recovery nutrition after high‐intensity exercise should be hierarchical and periodized. Carbohydrate and protein form the foundation for glycogen restoration and muscle repair, while functional foods and bioactive compounds serve as context‐dependent adjuncts. Selection should reflect recovery time, physiological stress, competition demands, and long‐term adaptation goals.

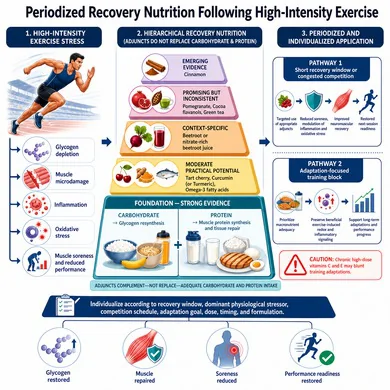

## 1 Introduction

High‐intensity exercise, including high‐intensity interval training (HIIT), sprint interval exercise, repeated‐sprint training, resistance exercise, eccentric exercise, and intermittent sport‐specific activity, is a central component of modern athletic conditioning (Chang et al. 2026). These training models are effective for improving aerobic and anaerobic capacity, power output, metabolic efficiency, and sport‐specific performance. Nevertheless, the same physiological stress that stimulates adaptation can also produce acute fatigue, glycogen depletion, exercise‐induced muscle damage, delayed‐onset muscle soreness, inflammatory signaling, oxidative stress, and transient impairment of subsequent performance (Harty et al. 2019; Leite et al. 2023; Naderi et al. 2025; Owens et al. 2019). In athletes exposed to congested training or competition schedules, the capacity to restore physiological readiness between sessions is therefore a decisive component of performance preparation.

Postexercise recovery is not a single biological process. It involves the restoration of energy stores, repair and remodeling of damaged muscle tissue, rehydration, resolution or regulation of inflammation, restoration of redox balance, and recovery of neuromuscular function. Exercise‐induced muscle damage is commonly characterized by increased creatine kinase, lactate dehydrogenase, myoglobin, delayed‐onset muscle soreness, reduced maximal voluntary contraction, impaired range of motion, and decreased jump, sprint, or strength performance. Furthermore, high‐intensity exercise may also acutely elevate inflammatory and redox‐related markers, including C‐reactive protein, interleukin‐6, tumor necrosis factor‐alpha, malondialdehyde, superoxide dismutase, glutathione peroxidase, and total antioxidant capacity (Harty et al. 2019; Leite et al. 2023; Owens et al. 2019).

Although these exercise modalities are collectively described as high‐intensity exercise, they differ substantially in the primary physiological stressors they impose. Repeated‐sprint and sprint interval exercise predominantly challenge phosphocreatine turnover, glycolytic metabolism, and rapid glycogen utilization (Lan et al. 2025), whereas resistance and eccentric exercise are characterized by greater mechanical loading and a higher propensity for exercise‐induced muscle damage and delayed‐onset muscle soreness (Boyd et al. 2023). Intermittent team‐sport activities combine repeated high‐intensity efforts with prolonged activity, resulting in concurrent metabolic and mechanical stress. Consequently, the relative importance of glycogen restoration, muscle protein synthesis, inflammation, oxidative stress, and neuromuscular recovery varies across exercise modalities. Throughout this review, these exercise models are considered within a unified recovery‐nutrition framework because they share the need for nutritional strategies that support recovery after substantial physiological stress, while acknowledging that the magnitude and timing of recovery demands are context‐dependent.

Sports nutrition has traditionally emphasized energy availability, macronutrient intake, hydration, and ergogenic supplementation. However, contemporary recovery nutrition extends beyond acute performance enhancement. Carbohydrate and protein remain the most established recovery nutrients because carbohydrate supports glycogen restoration and protein supports muscle protein synthesis and tissue repair (Craven et al. 2021; Papadopoulou 2020). Beyond these foundational strategies, functional foods and bioactive compounds are increasingly investigated for their potential effects on inflammation, oxidative stress, soreness, vascular function, muscle damage, and recovery of neuromuscular performance (Tkaczenko and Kurhaluk 2025; Wang et al. 2024).

The term functional food generally refers to foods or food‐derived components that provide physiological benefits beyond basic nutritional value (Roberfroid 2002). In this review, the term is used alongside bioactive compounds to accommodate both whole‐food interventions and isolated or concentrated compounds commonly used in sports nutrition research. In sports nutrition, this includes polyphenol‐rich foods and bioactives such as tart cherry, pomegranate, beetroot, cocoa flavanols, green tea catechins, curcumin, cinnamon, omega‐3 fatty acids, and antioxidant vitamins. However, the current evidence is heterogeneous. Some interventions improve functional outcomes without clear biomarker changes, whereas others alter biomarkers without improving strength, sprint performance, soreness, or next‐day readiness (Hagele et al. 2026; Mănescu et al. 2026; Suzuki et al. 2025). Moreover, inflammation and oxidative stress should not always be interpreted as harmful, because both redox and inflammatory signals contribute to exercise adaptation.

This creates an important conceptual and methodological gap. Existing reviews have commonly examined sports nutrition broadly or evaluated isolated supplements and functional foods separately. However, fewer reviews have integrated foundational macronutrient strategies with functional foods and bioactive compounds within a recovery‐centered and periodized framework after high‐intensity exercise. The novelty of this review lies in its hierarchical interpretation of recovery nutrition. Carbohydrate and protein are positioned as foundational strategies for glycogen restoration and muscle repair, whereas functional foods and bioactive compounds are interpreted as adjunct strategies that should be selected according to the recovery window, dominant physiological stressor, competition schedule, and adaptation goals. The present review extends this literature by integrating foundational macronutrient strategies with functional foods and bioactive compounds within a single recovery‐oriented framework. Rather than proposing new efficacy rankings, the review synthesizes current evidence into a hierarchical and periodized conceptual model that prioritizes nutritional interventions according to the dominant physiological stressor, practical recovery objective, and the relative strength of the available evidence.

The primary research question guiding this review was: Which nutritional strategies, ranging from foundational macronutrients to functional foods and bioactive compounds, have evidence for supporting biochemical, perceptual, and functional recovery following high‐intensity exercise in athletes and physically active adults? Secondary questions addressed, (i) the relative strength of evidence for foundational macronutrients and adjunct functional foods across different recovery outcomes; (ii) how functional foods and bioactive compounds should be interpreted within the recovery–adaptation paradigm; and (iii) the methodological limitations and research gaps that influence the translation of current evidence into applied sports nutrition practice. Therefore, this review aimed to synthesize current evidence on nutritional strategies for recovery following high‐intensity exercise, with emphasis on carbohydrate, protein, functional foods, bioactive compounds, inflammation, oxidative stress, muscle damage, soreness, and subsequent performance.

## 2 Materials and Methods

### 2.1 Review Design

This article was designed as a structured narrative review. This design was selected because the topic includes heterogeneous interventions, exercise models, populations, dosing regimens, supplementation durations, and recovery outcomes. A full meta‐analysis was not conducted because the evidence spans multiple nutritional categories and because substantial heterogeneity exists in exercise protocols, timing of biomarker assessment, supplement formulation, and performance‐recovery outcomes. Nevertheless, the search and synthesis process was structured to improve transparency, reproducibility, and methodological rigor, although the review was not designed as a formal PRISMA‐based systematic or scoping review.

### 2.2 Information Sources and Search Period

A structured literature search was conducted across PubMed/MEDLINE, Scopus, Web of Science, SPORTDiscus, ScienceDirect, and Google Scholar. The literature search covered articles published between January 2011 and May 2026. The starting year was selected to capture contemporary evidence reflecting the rapid expansion of research on sports nutrition, functional foods, bioactive compounds, and recovery science, while maintaining relevance to current evidence‐based practice. Earlier publications were included selectively when they represented foundational evidence or influential studies necessary to contextualize contemporary findings. The literature search was last updated in May 2026 to capture relevant recently published evidence available at the time of manuscript preparation.

### 2.3 Search Strategy

Search terms were developed around four conceptual blocks: exercise stress, nutritional intervention, recovery outcomes, and physiological markers. The core search logic combined terms related to high‐intensity exercise, recovery, sports nutrition, functional foods, bioactive compounds, and recovery biomarkers. Representative search strings are presented in Table 1.

**TABLE 1:** Structured search strategy used to identify relevant literature.

Database	Search string/search logic	Filters	Search date
PubMed/MEDLINE	(“high‐intensity exercise” OR HIIT OR “sprint interval training” OR “repeated sprint” OR “resistance exercise” OR “eccentric exercise” OR “exercise‐induced muscle damage”) AND (recovery OR “post‐exercise recovery” OR “muscle soreness” OR “muscle damage” OR “subsequent performance”) AND (nutrition OR carbohydrate OR protein OR “functional food” OR “bioactive compounds” OR polyphenols OR “tart cherry” OR beetroot OR pomegranate OR curcumin OR cinnamon OR omega‐3)	English; Human; 2011–2026	May 2026
Scopus	TITLE‐ABS‐KEY ((“high‐intensity exercise” OR HIIT OR “exercise‐induced muscle damage”) AND (recovery OR soreness OR inflammation OR “oxidative stress”) AND (“sports nutrition” OR carbohydrate OR protein OR “functional food” OR polyphenols OR curcumin OR beetroot OR “tart cherry”))	Article/Review; English; 2011–2026	May 2026
Web of Science	TS = ((“high‐intensity exercise” OR HIIT OR “sprint interval training” OR “resistance exercise”) AND (recovery OR “muscle damage” OR soreness OR inflammation OR “oxidative stress”) AND (nutrition OR “functional food” OR “bioactive compounds” OR polyphenols OR curcumin OR omega‐3))	English; 2011–2026	May 2026
SPORTDiscus	(“high‐intensity exercise” OR HIIT OR sprint OR resistance OR eccentric) AND (recovery OR soreness OR “muscle damage”) AND (nutrition OR carbohydrate OR protein OR “functional food” OR polyphenol OR curcumin OR beetroot OR “tart cherry”)	English; peer‐reviewed; 2011–2026	May 2026
ScienceDirect	(“high‐intensity exercise” OR HIIT OR “exercise‐induced muscle damage”) AND (“sports nutrition” OR “functional food” OR polyphenols OR protein OR carbohydrate) AND (recovery OR inflammation OR “oxidative stress”)	Research Articles/Reviews; English; 2011–2026	May 2026
Google Scholar	Supplementary citation tracking using combinations of “exercise recovery”, “functional food”, “sports nutrition”, “tart cherry”, “curcumin”, “beetroot”, “omega‐3”, “muscle damage”, and “oxidative stress”	Used only for citation tracking and identification of highly cited or recently published studies not retrieved from main databases	May 2026

Google Scholar was used only as a supplementary source for backward and forward citation tracking and to identify highly cited or recently published studies not retrieved from the main databases. It was not treated as the primary database because of limited reproducibility and less transparent indexing.

### 2.4 Eligibility Criteria

Studies were considered eligible when they involved athletes, trained individuals, or physically active adults and examined recovery following high‐intensity interval exercise, repeated‐sprint exercise, sprint interval exercise, resistance exercise, eccentric exercise, intermittent sport activity, or recognized exercise‐induced muscle damage protocols. Eligible interventions included nutritional strategies, functional foods, or bioactive compounds relevant to postexercise recovery. Studies were included when outcomes addressed glycogen restoration, muscle protein synthesis, muscle damage, inflammation, oxidative stress, muscle soreness, neuromuscular function, or subsequent exercise performance. Only peer‐reviewed articles published in English were considered. Priority was given to position stands, consensus statements, systematic reviews, meta‐analyses, randomized controlled trials, and human intervention studies relevant to exercise recovery.

Studies were excluded if they focused primarily on clinical disease populations without an exercise‐recovery context, animal or cell models lacking direct translational relevance to humans, gut microbiome‐centered interventions without recovery outcomes, weight‐loss dietary interventions unrelated to exercise recovery, or opinion‐based articles lacking a clear empirical or theoretical foundation.

### 2.5 Literature Screening, Selection, and Data Extraction

Search results were screened for relevance to the predefined review questions and eligibility criteria described above. Because this manuscript was conceived as a structured narrative review rather than a systematic or scoping review, database‐specific retrieval counts, duplicate‐removal counts, and reasons for exclusion at each screening stage were not prospectively recorded. Consequently, these numbers could not be reconstructed retrospectively with sufficient reliability, and we therefore did not generate post hoc numerical estimates or a PRISMA‐style study‐flow diagram. Nevertheless, the final manuscript cites 51 sources overall, comprising evidence directly informing the recovery‐nutrition synthesis as well as a small number of foundational or contextual references. Older publications outside the primary search period were retained only when they represented foundational evidence necessary to define concepts or contextualize contemporary findings.

Literature identification and initial eligibility assessment were conducted by H.R.F. and G.E.V., with methodological oversight by F.N. Evidence interpretation and the consistency of the resulting narrative synthesis were subsequently reviewed by A.S. and F.N. Any uncertainties regarding the relevance or interpretation of individual sources were resolved through discussion among the authors. Extracted information included author and year, evidence type, population, exercise model, intervention, dose and timing when available, recovery outcomes, key findings, and evidence interpretation. The extracted evidence was organized according to nutritional category and dominant recovery outcome to support the mechanism‐based narrative synthesis.

### 2.6 Evidence Appraisal and Evidence Grading

Evidence interpretation considered several complementary criteria rather than a single methodological characteristic. These included study design (e.g., position stands, consensus statements, systematic reviews, meta‐analyses, randomized controlled trials, and primary intervention studies), consistency of findings across independent studies, methodological quality, sample size, relevance of the exercise model and study population, clarity of supplementation dose, timing and duration, and the applicability of findings to sports practice. Evidence was classified qualitatively into four levels to improve transparency of the narrative synthesis. Strong evidence was assigned when findings were supported by high‐level evidence (position stands, consensus statements, meta‐analyses, or multiple well‐conducted randomized controlled trials) demonstrating consistent outcomes with clear practical relevance. Moderate evidence referred to findings supported by systematic reviews or several human intervention studies but with important heterogeneity in study design, participant characteristics, exercise protocols, supplementation strategies, or recovery outcomes. Low evidence indicated limited or inconsistent findings derived from a small number of studies, studies with methodological limitations, or evidence showing disagreement between biochemical and functional recovery outcomes. Very low evidence referred to preliminary findings supported by only a few exploratory studies, indirect evidence, or interventions for which exercise‐recovery‐specific data remain insufficient. This qualitative framework was developed solely to facilitate transparent interpretation of the narrative synthesis and should not be interpreted as a formal GRADE assessment or a quantitative evaluation of certainty of evidence. The qualitative evidence levels were intended to provide a structured interpretation of the literature rather than an objective scoring system or formal certainty‐of‐evidence assessment.

## 3 Results

### 3.1 Results and Evidence Synthesis

#### 3.1.1 Physiological Stress Following High‐Intensity Exercise

High‐intensity exercise produces a complex recovery burden because it challenges metabolic, mechanical, inflammatory, and redox systems simultaneously. During repeated high‐intensity efforts, skeletal muscle relies heavily on phosphocreatine breakdown, anaerobic glycolysis, and rapid glycogen utilization. When recovery time is short, incomplete glycogen restoration can reduce the ability to reproduce high‐intensity work in the next session (Burke et al. 2017; Craven et al. 2021; Kerksick et al. 2017; Ranchordas et al. 2017). Sprinting, resistance exercise, eccentric contractions, and intermittent sport‐specific activity can also produce muscle membrane disruption, excitation‐contraction coupling impairment, sarcomere damage, and delayed functional decline (Harty et al. 2019; Leite et al. 2023; Owens et al. 2019).

A systematic review on exercise‐induced muscle damage after a single high‐intensity interval exercise session reported that creatine kinase, myoglobin, and lactate dehydrogenase are among the most frequently used markers, with increases commonly observed immediately after exercise and during the first 24 h of recovery (Leite et al. 2023). Broader reviews describe exercise‐induced muscle damage as a multidimensional response involving soreness, force loss, local inflammation, oxidative stress, and reduced exercise capacity that may persist for several days depending on the intensity, novelty, and eccentric component of exercise (Harty et al. 2019; Owens et al. 2019). The biomarkers and functional outcomes most commonly used in exercise recovery research are summarized in Table 2.

**TABLE 2:** Biomarkers commonly used in exercise recovery studies.

Biomarker/outcome	Physiological meaning	Relevance to recovery	Interpretation caveat
CK	Muscle membrane disruption	Common indirect marker of exercise‐induced muscle damage	High interindividual variability; may not reflect functional recovery
LDH	Tissue and muscle stress	Supports interpretation of muscle damage and tissue stress	Less muscle‐specific than CK
Myoglobin	Muscle protein leakage	Acute marker of muscle damage	Timing‐sensitive; rises and clears rapidly
CRP	Systemic inflammation	Reflects delayed systemic inflammatory response	Nonspecific; affected by illness, adiposity, and baseline inflammation
IL‐6	Inflammatory and metabolic signaling	Reflects acute exercise stress and cytokine response	Can have both pro‐ and anti‐inflammatory roles depending on context
TNF‐α	Pro‐inflammatory cytokine	May indicate persistent inflammatory burden	Often unchanged in supplementation trials
MDA	Lipid peroxidation	Marker of oxidative damage	Assay variability matters; should be interpreted with antioxidant markers
SOD	Antioxidant enzyme activity	Reflects endogenous antioxidant defense	Increase may indicate oxidative stress or adaptive response
GPx	Peroxide detoxification	Reflects redox defense capacity	Should be interpreted alongside oxidative‐damage markers
TAC	Overall antioxidant capacity	Global indicator of antioxidant status	Nonspecific composite measure; does not identify specific pathways
DOMS	Perceived muscle soreness	Practical subjective recovery indicator	Influenced by expectation, training history, and pain tolerance
MVC	Maximal force production	Direct indicator of neuromuscular functional recovery	More sport‐relevant than biomarkers, but protocol‐dependent
CMJ	Explosive lower‐body performance	Practical marker of neuromuscular readiness	Sensitive to fatigue, motivation, and testing standardization
Sprint/repeated‐sprint performance	High‐intensity functional capacity	Directly relevant to sport performance and next‐session readiness	May not align with biochemical marker changes

Abbreviations: CK, creatine kinase; CMJ, countermovement jump; CRP, C‐reactive protein; DOMS, delayed‐onset muscle soreness; GPx, glutathione peroxidase; IL‐6, interleukin‐6; LDH, lactate dehydrogenase; MDA, malondialdehyde; MVC, maximal voluntary contraction; SOD, superoxide dismutase; TAC, total antioxidant capacity; TNF‐α, tumor necrosis factor‐alpha.

Throughout Section 3, biochemical markers (e.g., CK, LDH, myoglobin, CRP, IL‐6, TNF‐α, MDA, SOD, GPx, TAC) and functional/performance outcomes (e.g., MVC, CMJ, DOMS, sprint or repeated‐sprint performance) are reported and interpreted as distinct categories of evidence. As shown in the Interpretation Caveat column of Table 2, biochemical changes are indirect physiological signals and do not necessarily correspond to meaningful improvements in muscle function, soreness, or subsequent exercise performance; for each functional food discussed below, biomarker findings and functional/performance findings are therefore presented separately rather than treated as interchangeable indicators of “recovery.”

#### 3.1.2 Carbohydrate‐Based Strategies for Glycogen Restoration

Carbohydrate intake remains the most established nutritional strategy for restoring performance capacity when the recovery window between high‐intensity sessions is short. The International Society of Sports Nutrition position stand on nutrient timing recommends rapid carbohydrate provision when glycogen restoration is urgent, particularly when < 4 h separates exercise sessions (Kerksick et al. 2017). In such contexts, carbohydrate intake of approximately 1.0–1.2 g/kg/h during the early postexercise period is frequently recommended, with high‐glycemic index sources often preferred because they support faster glycogen resynthesis (Burke et al. 2017; Kerksick et al. 2017; Ranchordas et al. 2017).

Burke et al. (2017) emphasized that postexercise muscle glycogen resynthesis is strongly influenced by the timing, amount, and type of carbohydrate consumed. Immediate carbohydrate intake is most important when athletes must train or compete again within the same day. When the recovery period is longer, total daily carbohydrate intake becomes more important than immediate timing alone. This distinction is highly relevant for athletes in tournament formats, team sports, combat sports, and repeated daily training schedules.

The co‐ingestion of carbohydrate and protein has received substantial attention. Craven et al. (2021) reported that carbohydrate plus protein can increase muscle glycogen resynthesis when added protein increases total energy intake or when carbohydrate intake is suboptimal. However, when carbohydrate intake is already sufficient, adding protein does not necessarily further accelerate glycogen resynthesis. Collectively, the available evidence indicates that carbohydrate is the principal nutritional strategy for glycogen restoration, whereas protein co‐ingestion may further support recovery under specific nutritional conditions (Burke et al. 2017; Craven et al. 2021; Jäger et al. 2017; Kerksick et al. 2017; Pasiakos et al. 2014; Ranchordas et al. 2017).

#### 3.1.3 Protein and Amino Acids for Muscle Repair

Protein is the foundational nutrient for muscle repair and remodeling following high‐intensity and muscle‐damaging exercise. The International Society of Sports Nutrition position stand on protein and exercise recommends approximately 1.4–2.0 g/kg/day of protein for most exercising individuals, with higher intakes potentially relevant during energy restriction, high training load, or body composition manipulation (Jäger et al. 2017). A practical strategy is to distribute high‐quality protein across the day in servings of approximately 20–40 g, ideally containing sufficient essential amino acids and leucine to stimulate muscle protein synthesis (Jäger et al. 2017). Pasiakos et al. (2014) reported that protein supplementation can attenuate muscle damage and soreness while improving recovery of muscle function and physical performance after damaging exercise. However, the magnitude of benefit depends on baseline protein intake, training status, exercise type, protein quality, timing, and total daily intake.

Whey protein is frequently emphasized because it is rapidly digested and rich in leucine, an amino acid involved in activating muscle protein synthesis through mTORC1‐related signaling. Casein may be useful before sleep because of its slower digestion and potential to support overnight muscle protein synthesis (Jäger et al. 2017). Whole‐food protein sources such as milk, yogurt, eggs, fish, lean meat, legumes, and soy‐based foods can also serve as practical recovery foods when they provide high‐quality amino acids and adequate total protein.

#### 3.1.4 Tart Cherry as a Polyphenol‐Rich Recovery Food

Tart cherry is one of the most frequently studied functional foods for postexercise recovery. It contains anthocyanins, phenolic acids, and other polyphenols with potential antioxidant and anti‐inflammatory properties. Proposed mechanisms include reduced cyclooxygenase activity, attenuation of oxidative damage, modulation of inflammatory signaling, and improved recovery of muscle function. Hill et al. (2021) reported that tart cherry supplementation may support recovery from strenuous exercise, although effects varied according to exercise model, supplementation duration, and outcome measure. More recent synthesis in trained athletes suggested significant improvements in maximal voluntary contraction recovery and reductions in C‐reactive protein at selected postexercise time points, while pooled effects on soreness, creatine kinase, interleukin‐6, tumor necrosis factor‐alpha, and range of motion were less consistent (Daab et al. 2026). Across the trials pooled by Hill et al. (2021), tart cherry was typically consumed as juice or concentrate at one to two servings per day for 7–16 days, spanning several days before, the day of, and several days after exercise; this variability in dose, formulation, and supplementation window likely contributes to the inconsistent effects noted above.

These findings indicate that tart cherry should not be interpreted as a universal suppressor of all muscle‐damage and inflammatory markers. Its strongest evidence appears to relate to selected functional outcomes and some inflammatory responses, particularly when consumed before and after damaging exercise. In applied practice, tart cherry may be most useful during congested competition, tournament play, or repeated high‐intensity sessions where rapid restoration of muscle function is more important than maximizing long‐term adaptive signaling.

#### 3.1.5 Pomegranate and Exercise Recovery

Pomegranate contains ellagitannins, punicalagins, anthocyanins, and other polyphenols with antioxidant and anti‐inflammatory potential. Ammar et al. (2018) concluded that pomegranate supplementation may enhance exercise performance and postexercise recovery in healthy adults, particularly when products are rich in polyphenols and consumed with sufficient timing before exercise. However, more recent meta‐analytic evidence suggests that pomegranate supplementation does not consistently improve creatine kinase, myoglobin, lactate, or soreness, although it may reduce lactate dehydrogenase immediately after exercise (Belyani et al. 2025).

The inconsistency may be explained by variation in pomegranate form, dose, polyphenol concentration, timing, participant training status, and exercise model. Therefore, pomegranate should be considered a promising but lower‐certainty functional food for exercise recovery compared with tart cherry. Its potential effect may be more relevant to short‐term mechanical muscle‐damage responses than broad performance recovery. Reported protocols range from single pre‐exercise doses to sustained supplementation for up to 2 weeks, using juice, extract, or concentrate formulations that differ substantially in polyphenol content; this heterogeneity in dose, formulation, and timing limits direct comparison across studies (Ammar et al. 2018; Belyani et al. 2025).

#### 3.1.6 Beetroot and Nitrate‐Related Recovery

Beetroot is typically studied for its nitrate content, which can increase nitric oxide bioavailability and influence blood flow, mitochondrial efficiency, oxygen delivery, and muscle contractile function. Beetroot also contains betalains and other antioxidant compounds, which may contribute to redox modulation.

Rojano‐Ortega et al. (2022) found that beetroot supplementation may improve functional recovery and reduce muscle soreness after exercise‐induced muscle damage, although effects on muscle‐damage, inflammatory, and oxidative‐stress biomarkers were inconsistent. Jones et al. (2022) similarly suggested that nitrate‐rich beetroot juice may attenuate selected markers of exercise‐induced muscle damage, but the evidence remains heterogeneous. Across the beetroot literature, protocols typically provide dietary nitrate as juice or concentrate, consumed either acutely 2–3 h before exercise or repeatedly for several days before and after exercise; nitrate dose and product formulation vary considerably across studies, which limits direct comparability (Jones et al. 2022; Rojano‐Ortega et al. 2022).

Primary studies support context‐dependent effects. Clifford, Bell, et al. (2016) reported that beetroot juice attenuated muscle soreness and decrements in countermovement jump performance after eccentric exercise. Another study showed that beetroot juice improved recovery of muscle function and performance between repeated‐sprint bouts (Clifford, Berntzen, et al. 2016). However, beetroot juice did not meaningfully affect inflammation and recovery after a marathon when muscle damage was minimal (Clifford et al. 2017). Collectively, beetroot appears more convincing for soreness and functional recovery under specific conditions than for consistent modulation of inflammatory or muscle‐damage biomarkers.

#### 3.1.7 Cocoa Flavanols and Redox‐Related Recovery

Cocoa contains flavanols such as epicatechin and catechin, which may influence endothelial function, nitric oxide availability, antioxidant capacity, and vascular responses to exercise. Decroix et al. (2018) suggested that cocoa flavanol supplementation may influence exercise performance and recovery through antioxidant, anti‐inflammatory, and vascular pathways. However, later reviews indicate that evidence for direct improvements in soreness, muscle function, and performance recovery remains inconsistent (Corr et al. 2021; Massaro et al. 2019).

Corr et al. (2021) concluded that cocoa flavanols appear more consistently associated with reduced exercise‐induced oxidative stress than with clear improvements in muscle recovery or performance. Massaro et al. (2019) similarly noted that cocoa products may reduce oxidative stress but have less consistent effects on inflammation and exercise‐induced muscle damage. Therefore, cocoa flavanols should be positioned as redox‐supportive functional foods rather than robust recovery‐enhancing interventions. Reported cocoa flavanol protocols vary in flavanol content, product form (dark chocolate, cocoa extract, or beverage), and duration, ranging from acute pre‐exercise intake to several weeks of daily supplementation; this heterogeneity further limits comparability across studies (Corr et al. 2021; Decroix et al. 2018; Massaro et al. 2019).

#### 3.1.8 Green Tea Catechins and Oxidative‐Stress Modulation

Green tea is rich in catechins, particularly epigallocatechin gallate, which may exert antioxidant and anti‐inflammatory effects. Rojano‐Ortega (2021) concluded that regular green tea supplementation, but not acute ingestion, may increase total antioxidant status and reduce exercise‐induced oxidative stress. Supplementation periods longer than 1 week and catechin doses around 400–800 mg/day appear more plausible for redox‐related effects (Rojano‐Ortega 2021).

Jówko et al. (2015) reported favorable effects of green tea extract supplementation on oxidative‐stress parameters in male sprinters. Machado et al. (2018) also found that green tea extract preserved neuromuscular activation and reduced markers of muscle damage in athletes experiencing cumulative fatigue. Nevertheless, the evidence remains limited and does not consistently demonstrate improvements in soreness or subsequent performance. Green tea catechins may therefore be considered potentially useful for oxidative‐stress regulation during repeated high‐intensity training, but not as a primary recovery strategy.

#### 3.1.9 Curcumin and Exercise‐Induced Inflammation

Curcumin, the primary bioactive compound in turmeric, has received substantial attention because of its anti‐inflammatory and antioxidant potential. Proposed mechanisms include modulation of nuclear factor‐kappa B, cyclooxygenase pathways, cytokine production, oxidative stress, and muscle‐damage responses.

Fernández‐Lázaro et al. (2020) concluded that curcumin supplementation may modulate exercise‐induced muscle damage, inflammation, and oxidative markers in physically active populations. Ms et al. (2020) reported that curcumin may reduce muscle damage and perceived soreness without clearly suppressing the natural inflammatory response. Later reviews suggested that curcumin may reduce soreness intensity and creatine kinase responses across a broad range of doses, but findings remain influenced by supplementation timing, dose, duration, and formulation (Daniel Vasile et al. 2024; Nanavati et al. 2022).

A major limitation is bioavailability. Standard oral curcumin has relatively poor absorption, and many formulations use piperine, phospholipid complexes, nanoparticles, or other delivery systems to enhance bioavailability (Tabanelli et al. 2021). This creates translational difficulty because “curcumin” studies are not necessarily comparable across products. Safety also requires attention, particularly for concentrated turmeric or curcumin supplement products and enhanced‐bioavailability formulations (Halegoua‐DeMarzio et al. 2023). For sports nutrition practice, curcumin may be a promising adjunct during periods of high muscle soreness or repeated damaging exercise, but recommendations should specify formulation, dose, timing, and safety considerations. Reported protocols typically use curcumin doses of approximately 150–1500 mg/day, initiated before exercise and continued for up to 72 h postexercise, although formulation‐dependent bioavailability differences make cross‐study comparison difficult (Fernández‐Lázaro et al. 2020).

#### 3.1.10 Cinnamon as an Emerging Bioactive Compound

Cinnamon contains cinnamaldehyde, cinnamic acid, and polyphenolic compounds that may influence antioxidant activity, inflammation, glucose metabolism, and pain perception. From a sports nutrition perspective, cinnamon is of interest because of its potential metabolic and anti‐inflammatory properties. However, evidence directly linking cinnamon to exercise recovery remains limited.

Studies in Iranian female athletes reported that cinnamon or ginger intake did not significantly improve malondialdehyde, body composition, or exercise performance compared with placebo, and did not show strong between‐group improvements in inflammatory markers (Mashhadi, Ghiasvand, Askari, et al. 2013; Mashhadi, Ghiasvand, Hariri, et al. 2013). Some modest signals for reduced soreness were reported, but the evidence was small, population‐specific, and not sufficient for strong recommendations. Cinnamon should therefore be classified as an emerging bioactive compound with very low certainty in exercise recovery. Precise dose, formulation, and supplementation duration were not consistently reported across the available trials, which further constrains interpretation (Mashhadi, Ghiasvand, Askari, et al. 2013; Mashhadi, Ghiasvand, Hariri, et al. 2013).

#### 3.1.11 Omega‐3 Fatty Acids and Recovery

Omega‐3 fatty acids, particularly eicosapentaenoic acid and docosahexaenoic acid, may support recovery through membrane‐related effects, modulation of inflammatory mediators, production of specialized pro‐resolving lipid mediators, and potential effects on muscle function and soreness.

Xin and Eshaghi (2021) reported significant reductions in indirect blood markers of exercise‐induced muscle damage, including creatine kinase, lactate dehydrogenase, and myoglobin, following omega‐3 supplementation. Fernández‐Lázaro et al. (2024) found that omega‐3 supplementation may improve some markers such as creatine kinase, lactate dehydrogenase, C‐reactive protein, and interleukin‐6, although findings for tumor necrosis factor‐alpha, delayed‐onset muscle soreness, oxidative stress, and performance outcomes remain heterogeneous. Heileson et al. (2024) showed that long‐chain omega‐3 supplementation, particularly eicosapentaenoic acid or docosahexaenoic acid separately, may improve aspects of soreness and strength recovery after damaging exercise. The International Society of Sports Nutrition position stand on long‐chain omega‐3 polyunsaturated fatty acids recognizes their potential relevance for exercise performance, recovery, and health while emphasizing variability in response (Jäger et al. 2025).

Overall, omega‐3 fatty acids have moderate but not definitive evidence for recovery. They appear most promising for attenuating muscle‐damage markers and selected inflammatory or functional outcomes. However, optimal dose, duration, EPA/DHA ratio, baseline omega‐3 status, and sport‐specific application remain unresolved. Across the cited studies, EPA and/or DHA doses ranged from approximately 0.5 to 4 g/day, with supplementation durations spanning one to several weeks (e.g., 4 g/day of EPA or DHA for 7 weeks in Heileson et al. 2024); this heterogeneity in dose, duration, and EPA/DHA ratio likely contributes to the inconsistent findings described above (Fernández‐Lázaro et al. 2024; Heileson et al. 2024; Xin and Eshaghi 2021).

#### 3.1.12 Vitamins C and E: Antioxidant Benefit Versus Adaptation Risk

Vitamins C and E are classical antioxidant nutrients that can reduce oxidative stress by scavenging free radicals and supporting redox balance. However, their role in athletes is controversial. Although vitamin C and E may reduce oxidative‐stress markers in some studies, evidence for meaningful improvements in recovery, soreness, or performance remains inconsistent (Rogers et al. 2023).

More importantly, chronic high‐dose supplementation may interfere with exercise‐induced adaptation. Paulsen et al. (2014) reported that vitamin C and E supplementation hampered some cellular adaptations to endurance training in humans. Morrison et al. (2015) similarly found that vitamin C and E supplementation prevented some cellular adaptations following endurance training. Peternelj and Coombes (2011) argued that antioxidant supplementation during exercise training may be beneficial in some contexts but detrimental in others, particularly when it suppresses redox signaling required for adaptation. The cautionary evidence for adaptation‐blunting is based on chronic high‐dose protocols, most notably 1000 mg/day vitamin C combined with 235 mg/day vitamin E for 10–11 weeks (Morrison et al. 2015; Paulsen et al. 2014), doses that substantially exceed typical dietary or food‐based antioxidant intake and should not be extrapolated to lower‐dose, food‐based approaches.

The practical implication is that high‐dose vitamin C and E supplementation should not be used routinely as a general recovery strategy for athletes. Food‐based antioxidant intake from fruits, vegetables, and polyphenol‐rich foods is more defensible than chronic pharmacological‐dose supplementation unless deficiency, clinical indication, or a specific competition context justifies use. A summary of key studies and evidence sources informing the present synthesis is presented in Table 3.

**TABLE 3:** Key studies and evidence sources informing the synthesis.

Study	Evidence type	Population	Exercise context	Intervention	Main outcomes	Key findings	Evidence level	Typical dose/duration/timing (as reported)
Kerksick et al. (2017)	ISSN position stand	Exercising individuals/athletes	Exercise recovery	Carbohydrate and protein timing	Glycogen, recovery, adaptation	Supports nutrient timing within total daily intake, especially rapid carbohydrate when recovery time is short	Strong	Rapid CHO (~1.0–1.2 g/kg/h) when < 4 h between sessions; timing‐focused position stand
Burke et al. (2017)	Applied physiology review	Exercising humans	Postexercise glycogen depletion	Carbohydrate amount, type, and timing	Muscle glycogen	Immediate carbohydrate intake is most relevant when another session occurs within the same day	Strong	CHO amount/type/timing reviewed generally; ~1.0–1.2 g/kg/h emphasized for same‐day recovery
Craven et al. (2021)	Systematic review/meta‐analysis	Exercising adults	Short‐term postexercise recovery	Carbohydrate with or without protein	Glycogen resynthesis	Protein enhances glycogen resynthesis mainly when carbohydrate is suboptimal or total energy increases	Strong/moderate	Protocols heterogeneous across pooled trials; dose/duration not uniformly reported
Jäger et al. (2017)	ISSN position stand	Exercising individuals/athletes	Resistance and endurance exercise	Protein intake and distribution	Muscle protein synthesis, adaptation	Supports 1.4–2.0 g/kg/day protein and repeated high‐quality protein servings	Strong	~1.4–2.0 g/kg/day protein, distributed in ~20–40 g servings
Pasiakos et al. (2014)	Systematic review	Physically active adults	Muscle‐damaging exercise	Protein supplementation	Muscle damage, soreness, function	Protein may attenuate soreness and support functional recovery after damaging exercise	Moderate‐to‐strong	Protein dose/timing varied across included trials; not uniformly standardized
Hill et al. (2021)	Systematic review/meta‐analysis	Healthy adults/athletes	Strenuous exercise	Tart cherry supplementation	Soreness, strength recovery, biomarkers	Tart cherry may support recovery, but effects vary by protocol and outcome	Moderate	1–2 servings/day for 7–16 days, spanning pre‐, during‐, and postexercise
Daab et al. (2026)	Systematic review/meta‐analysis	Trained athletes	Exercise‐induced muscle damage	Tart cherry juice	MVC, CRP, CK, IL‐6, TNF‐α	Improved selected MVC recovery and CRP, with inconsistent effects on other markers	Moderate	Tart cherry juice; dose/duration variable across pooled trials
Ammar et al. (2018)	Systematic review	Healthy adults	Exercise performance and recovery	Pomegranate juice/extract	Recovery and performance	Potential benefit depends on polyphenol content and timing	Low‐to‐moderate	Dose, formulation (juice/extract), and timing varied substantially across studies
Belyani et al. (2025)	Systematic review/meta‐analysis	Healthy/active adults	Exercise‐induced muscle damage	Pomegranate supplementation	CK, myoglobin, LDH, soreness	Limited broad benefit, with possible acute LDH reduction	Low‐to‐moderate	Ten pooled trials with heterogeneous dose, formulation, and duration
Rojano‐Ortega et al. (2022)	Systematic review	Active adults	Exercise‐induced muscle damage	Beetroot supplementation	Soreness, function, biomarkers	May improve soreness and function, but biomarker findings are inconsistent	Low‐to‐moderate	Nitrate dose and supplementation duration varied across included trials
Clifford, Bell, et al. (2016)	Human intervention study	Recreationally active males	Eccentric exercise	Beetroot juice	CMJ, soreness, muscle damage	Attenuated soreness and countermovement jump decrement	Context‐specific	Beetroot juice (dietary nitrate) before and after eccentric exercise; single‐bout protocol
Clifford, Berntzen, et al. (2016)	Human intervention study	Team‐sport/recreationally active participants	Repeated‐sprint exercise	Beetroot juice	Muscle function, repeated‐sprint performance	Improved recovery between repeated‐sprint bouts	Context‐specific	Beetroot juice (dietary nitrate) across repeated‐sprint sessions
Decroix et al. (2018)	Systematic review	Exercising adults	Exercise performance and recovery	Cocoa flavanols	Vascular, oxidative, performance outcomes	Mechanistically plausible, but recovery effects remain inconsistent	Low	Flavanol content, product form, and duration varied across included studies
Rojano‐Ortega (2021)	Systematic review	Exercising adults	Exercise‐induced oxidative stress	Green tea catechins	TAC, oxidative stress	Regular supplementation may improve antioxidant status; acute ingestion is less supported	Low‐to‐moderate	Regular supplementation typically > 1 week; catechin doses ~400–800 mg/day
Fernández‐Lázaro et al. (2020)	Systematic review	Physically active populations	Exercise‐induced muscle damage	Curcumin supplementation	EIMD, inflammation, oxidative markers	May modulate muscle damage and inflammation, but formulation varies	Moderate	~150–1500 mg/day, from before exercise up to 72 h postexercise
Daniel Vasile et al. (2024)	Systematic review	Athletes/active adults	Exercise‐induced muscle damage	Curcumin supplementation	CK, soreness, muscle damage	Supports selected EIMD outcomes, with bioavailability concerns	Moderate	Curcumin dose/formulation heterogeneous across included studies
Mashhadi, Ghiasvand, Askari, et al. (2013), Mashhadi, Ghiasvand, Hariri, et al. (2013)	Human intervention studies	Iranian female athletes	Exercise‐induced soreness/training	Cinnamon or ginger intake	MDA, soreness, performance, inflammation	Cinnamon evidence is limited and not clearly positive	Very low	Cinnamon or ginger intake; specific dose/duration not consistently reported
Xin and Eshaghi (2021)	Systematic review/meta‐analysis	Healthy/active adults	Exercise‐induced muscle damage	Omega‐3 fatty acids	CK, LDH, myoglobin	Reduced indirect muscle‐damage markers	Moderate	EPA/DHA dose and duration varied across pooled trials
Fernández‐Lázaro et al. (2024)	Systematic review of randomized controlled trials	Physically healthy adults	Exercise recovery	Omega‐3 fatty acids	Inflammation, muscle damage, oxidative stress, performance	Some markers improve, but outcomes remain heterogeneous	Moderate	EPA/DHA dose (~0.5–4 g/day) and duration (1 to several weeks) varied across included RCTs
Paulsen et al. (2014)	Randomized controlled trial	Recreationally trained adults	Endurance training	High‐dose vitamin C and E	Cellular adaptation	Hampered selected training adaptations	Cautionary	1000 mg/day vitamin C + 235 mg/day vitamin E for 11 weeks
Morrison et al. (2015)	Human trial	Healthy adults	Endurance training	High‐dose vitamin C and E	Cellular adaptation	Prevented some cellular adaptations to training	Cautionary	High‐dose vitamin C and E over several weeks; comparable to Paulsen et al. protocol

### 3.2 Integrated Synthesis

Although the reviewed interventions were evaluated across diverse exercise models, including endurance, repeated‐sprint, resistance, eccentric, and intermittent sport activities, their effectiveness should be interpreted within the specific physiological demands and recovery outcomes of each exercise context. Accordingly, direct generalization across exercise modalities should be made with caution. The available evidence supports a hierarchical and periodized model of recovery nutrition after high‐intensity exercise (Table 4). In this model, carbohydrate and protein represent foundational recovery strategies because their mechanisms are directly linked to the most reproducible recovery demands: glycogen restoration and skeletal muscle repair. The available evidence supports interpreting functional foods and bioactive compounds as adjunct strategies rather than substitutes for macronutrient adequacy. Their role is more context‐specific, targeting soreness, oxidative stress, inflammation, muscle damage, vascular function, or neuromuscular recovery.

**TABLE 4:** Hierarchy of evidence for nutritional recovery strategies following high‐intensity exercise.

Evidence category	Intervention	Interpretation	Relative evidence volume
Foundational evidence	Carbohydrate, protein	Strongest and most transferable evidence; should form the base of recovery nutrition for glycogen restoration and muscle repair	Multiple ISSN position stands plus dozens of RCTs; broad and consistent
Moderate practical potential	Tart cherry, curcumin, omega‐3 fatty acids	Useful adjuncts for soreness, selected inflammatory outcomes, muscle damage, or functional recovery; effects depend on timing, dose, and context	≥ 2 systematic reviews/meta‐analyses per intervention (~10–20 primary studies); moderate heterogeneity
Context‐specific	Beetroot	More convincing for soreness or neuromuscular recovery than consistent inflammatory or muscle‐damage biomarker changes	1 systematic review plus 2 primary RCTs; small volume, context‐dependent
Promising but inconsistent	Pomegranate, cocoa flavanols, green tea	More consistent for oxidative‐stress modulation than direct performance recovery	1–2 systematic reviews or ~3–10 primary trials per intervention; inconsistent
Very limited or emerging	Cinnamon	Biological plausibility exists, but exercise‐recovery evidence is insufficient	2 small primary studies in a single population; very limited
Use with caution	High‐dose vitamin C and E	May reduce oxidative stress but chronic high‐dose use may blunt training adaptation	2 RCTs; small volume but high methodological quality

Abbreviations: CK, creatine kinase; CMJ, countermovement jump; CRP, C‐reactive protein; EIMD, exercise‐induced muscle damage; IL‐6, interleukin‐6; LDH, lactate dehydrogenase; MDA, malondialdehyde; MVC, maximal voluntary contraction; TAC, total antioxidant capacity; TNF‐α, tumor necrosis factor‐alpha.

The volume and consistency of the underlying evidence differ markedly across categories in Table 4. Carbohydrate and protein recommendations are underpinned by International Society of Sports Nutrition position stands that synthesize dozens of randomized controlled trials with broadly consistent findings. Tart cherry, curcumin, and omega‐3 fatty acids are each supported by two or more systematic reviews or meta‐analyses summarizing approximately 10–20 primary studies, with moderate heterogeneity. Beetroot, pomegranate, cocoa flavanols, and green tea are each supported by only one or two systematic reviews or a small number (approximately 3–10) of primary trials, with substantial heterogeneity in outcomes. Cinnamon evidence derives from only two small primary studies conducted in a single population. This variation in the amount and consistency of available evidence, and not mechanistic plausibility alone, is what underlies the evidence levels assigned in Table 3 and the categories shown in Table 4.

This hierarchy also implies that nutritional strategies should be selected according to the dominant recovery limitation. When the primary limitation is rapid restoration of energy availability, carbohydrate intake is the priority. When the dominant limitation is muscle repair or exercise‐induced muscle damage, adequate protein intake and selected adjunct strategies may be more relevant. When the goal is short‐term readiness during congested competition, targeted use of tart cherry, curcumin, beetroot, or omega‐3 fatty acids may be justified. In contrast, when the goal is long‐term adaptation, aggressive antioxidant or anti‐inflammatory strategies should be used cautiously to avoid unnecessary suppression of redox and inflammatory signaling.

Across the reviewed evidence, tart cherry currently has one of the strongest profiles among polyphenol‐rich foods, particularly for selected functional and inflammatory outcomes. Curcumin is promising for soreness and muscle‐damage modulation, although its interpretation is formulation‐sensitive. Omega‐3 fatty acids have moderate but heterogeneous support, especially for muscle‐damage and inflammatory markers. Beetroot appears useful in selected neuromuscular recovery contexts, whereas pomegranate, cocoa flavanols, and green tea appear more relevant for oxidative‐stress modulation than for consistent performance recovery. Cinnamon remains preliminary and should be framed as an emerging compound with very low exercise‐recovery evidence.

A recurring methodological issue is that biomarker improvement does not necessarily indicate meaningful functional recovery. Reductions in creatine kinase, lactate dehydrogenase, C‐reactive protein, interleukin‐6, malondialdehyde, or other markers do not always translate into improved strength, sprint performance, countermovement jump, or next‐session readiness. Therefore, recovery nutrition research and applied interpretation should integrate biochemical markers with perceptual and functional outcomes.

## 4 Discussion

**FIGURE 1:**
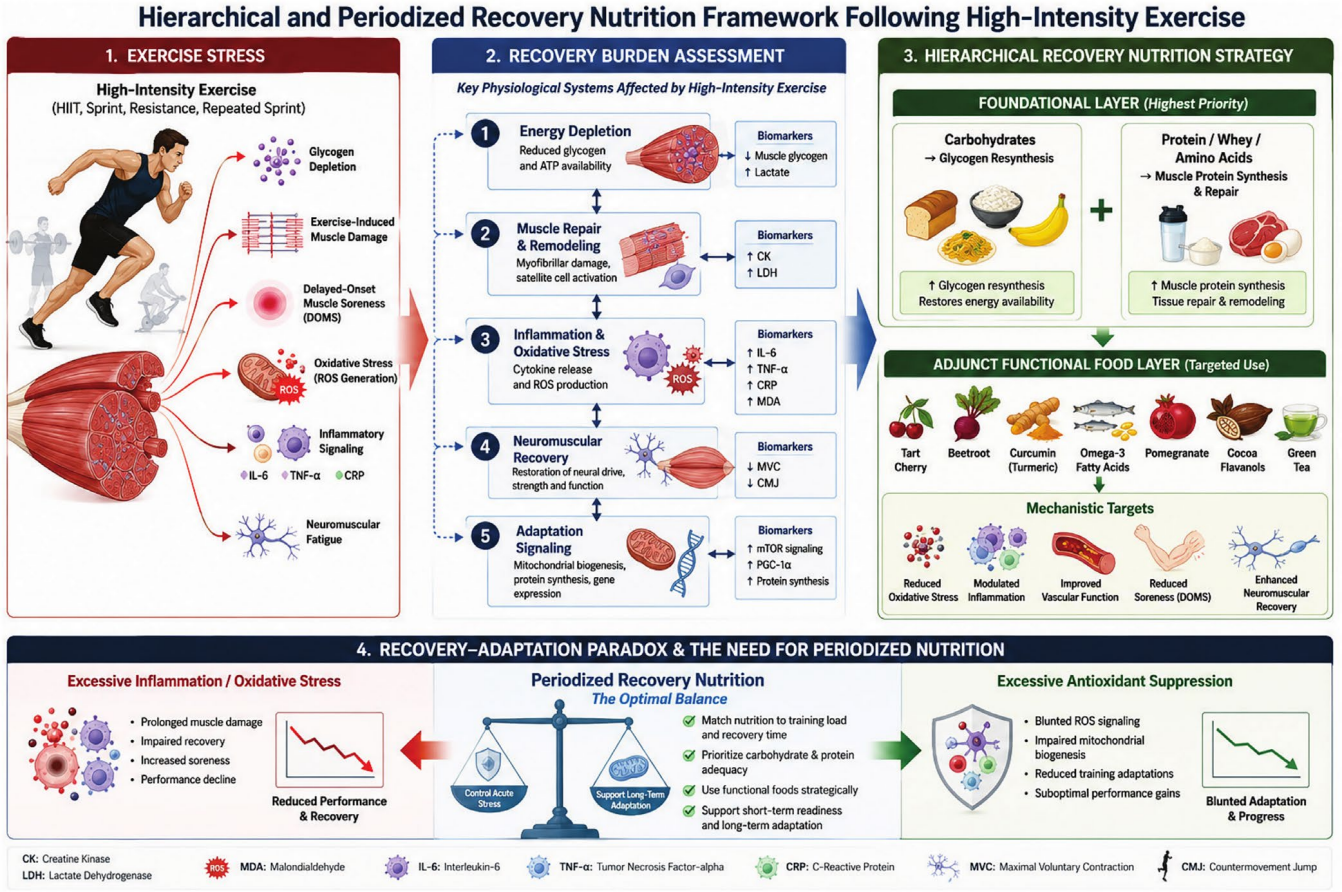
Hierarchical and periodized recovery nutrition framework following high‐intensity exercise. High‐intensity exercise induces a multifactorial physiological stress response characterized by glycogen depletion, exercise‐induced muscle damage, delayed‐onset muscle soreness (DOMS), oxidative stress, inflammatory signaling, and neuromuscular fatigue. These responses collectively influence recovery burden through disturbances in energy availability, skeletal muscle remodeling, redox balance, inflammatory regulation, and neuromuscular function. We propose that recovery nutrition may be interpreted through a hierarchical and periodized framework, recognizing that this represents an author‐derived conceptual synthesis requiring prospective validation. Functional foods and bioactive compounds, including tart cherry, beetroot, curcumin, omega‐3 fatty acids, pomegranate, cocoa flavanols, and green tea, may function as adjunct recovery strategies targeting oxidative stress, inflammation, vascular function, soreness, and neuromuscular recovery. The lower panel illustrates the recovery–adaptation paradox, emphasizing that both excessive inflammation and excessive antioxidant suppression may impair optimal adaptation. Therefore, nutritional recovery interventions should be periodized according to training load, recovery window, competition schedule, and long‐term adaptation goals. CK, creatine kinase; CMJ, countermovement jump; CRP, C‐reactive protein; IL‐6, interleukin‐6; LDH, lactate dehydrogenase; MDA, malondialdehyde; MVC, maximal voluntary contraction; ROS, reactive oxygen species; TNF‐α, tumor necrosis factor‐alpha.

Based on the integrated evidence synthesized in this review, we propose a hierarchical and periodized conceptual framework for recovery nutrition following high‐intensity exercise. Rather than treating all recovery foods, supplements, and bioactive compounds as equivalent, the reviewed evidence indicates that nutritional strategies may be prioritized according to the dominant recovery problem (including glycogen depletion, skeletal muscle repair, soreness, inflammation, oxidative stress, vascular function, and next‐session performance). Based on this synthesis, we propose a hierarchical approach to facilitate evidence‐informed decision‐making (Figure 1; Harty et al. 2019; Leite et al. 2023; Naderi et al. 2025; Owens et al. 2019). This perspective extends previous discussions of postexercise recovery by integrating foundational macronutrient strategies with functional foods and bioactive compounds within a recovery‐oriented decision framework. Importantly, the principal challenge in interpreting the current literature is not the absence of potentially effective interventions, but the considerable heterogeneity in exercise models, supplementation protocols, participant characteristics, and recovery outcomes, which limits direct comparison across studies and precludes universal recommendations.

An additional consideration is that the interventions included in the proposed framework operate across different temporal scales. Carbohydrate intake is primarily an acute postexercise strategy intended to accelerate glycogen resynthesis, whereas protein contributes both to immediate postexercise muscle protein synthesis and to cumulative daily protein adequacy. In contrast, several functional foods and bioactive compounds, including tart cherry, curcumin, and omega‐3 fatty acids, have most commonly been evaluated using short‐term preloading or repeated supplementation protocols extending over several days or weeks. Consequently, the proposed framework should not be interpreted as presenting these interventions as directly interchangeable recovery options, but rather as complementary strategies that differ in physiological targets, timing, and duration of application.

The first implication of this synthesis is that macronutrient adequacy remains the foundation of recovery nutrition. Carbohydrate and protein have the strongest and most transferable evidence because their mechanisms directly correspond to two major recovery demands: glycogen restoration and skeletal muscle repair (Burke et al. 2017; Craven et al. 2021; Jäger et al. 2017; Kerksick et al. 2017; Pasiakos et al. 2014; Ranchordas et al. 2017). Rapid postexercise carbohydrate provision is particularly relevant when recovery time between sessions is limited, whereas adequate daily carbohydrate intake becomes increasingly important when longer recovery windows are available (Burke et al. 2017; Craven et al. 2021; Kerksick et al. 2017; Ranchordas et al. 2017). Similarly, protein intake distributed throughout the day supports muscle protein synthesis, tissue remodeling, and attenuation of exercise‐induced muscle damage (Jäger et al. 2017; Pasiakos et al. 2014). Protein should therefore be interpreted as a daily recovery substrate, not merely as an immediate postexercise supplement. Functional foods and bioactive compounds may provide additional support; however, their practical effectiveness depends on whether foundational recovery practices, including sufficient energy intake, carbohydrate availability, protein distribution, hydration, and sleep, have already been optimized (Harty et al. 2019; Naderi et al. 2025). This distinction is especially relevant in applied sport settings, where specialized recovery supplements are often prioritized despite inadequate foundational nutritional practices.

The second implication is that functional foods and bioactive compounds should be interpreted as context‐specific adjuncts rather than universal recovery interventions. Among the polyphenol‐rich foods reviewed, tart cherry currently appears to demonstrate the clearest recovery profile, particularly for selected inflammatory and functional outcomes (Daab et al. 2026; Hill et al. 2021). Beetroot supplementation may be useful in repeated‐sprint or neuromuscular recovery contexts, especially where eccentric muscle damage contributes to soreness and reductions in explosive performance (Clifford et al. 2017; Clifford, Bell, et al. 2016; Clifford, Berntzen, et al. 2016; Jones et al. 2022; Rojano‐Ortega et al. 2022). In contrast, pomegranate, cocoa flavanols, and green tea appear more consistently associated with modulation of oxidative‐stress responses than with reproducible improvements in direct performance recovery (Ammar et al. 2018; Belyani et al. 2025; Corr et al. 2021; Decroix et al. 2018; Jówko et al. 2015; Machado et al. 2018; Massaro et al. 2019; Rojano‐Ortega 2021). This distinction is important because antioxidant activity alone should not be interpreted as evidence of meaningful recovery enhancement. Improvements in biochemical markers do not necessarily translate into practical improvements in soreness, maximal voluntary contraction, countermovement jump performance, sprint ability, or next‐session readiness (Harty et al. 2019; Leite et al. 2023; Owens et al. 2019). Therefore, recovery interventions should be interpreted through an integrated lens that considers biochemical, perceptual, and functional outcomes simultaneously.

An important consideration when interpreting the current evidence is that biochemical recovery does not necessarily equate to functional recovery. Although reductions in inflammatory cytokines or oxidative stress markers may indicate modulation of physiological responses to exercise, these changes do not consistently translate into improvements in muscle strength, sprint performance, countermovement jump performance, or subsequent exercise capacity. Likewise, some interventions may improve perceptual or functional recovery despite producing minimal changes in conventional biomarkers. Consequently, recovery strategies should be evaluated using an integrated framework that considers biochemical, perceptual, and functional outcomes collectively rather than relying on individual biomarkers as surrogate indicators of recovery effectiveness.

Curcumin and omega‐3 fatty acids may be particularly relevant when the dominant recovery burden involves soreness, muscle damage, or inflammatory responses. However, translation into applied sport settings remains constrained by substantial heterogeneity in formulation, dose, duration, timing, and participant characteristics. Curcumin is especially formulation‐sensitive because conventional oral curcumin exhibits poor bioavailability, whereas formulations incorporating piperine, phospholipid complexes, or nanoparticle delivery systems may substantially alter physiological effects (Daniel Vasile et al. 2024; Fernández‐Lázaro et al. 2020; Halegoua‐DeMarzio et al. 2023; Ms et al. 2020; Nanavati et al. 2022; Tabanelli et al. 2021). Moreover, recent concerns regarding hepatotoxicity associated with concentrated turmeric products reinforce the importance of cautious interpretation of high‐dose supplementation (Halegoua‐DeMarzio et al. 2023). Similarly, omega‐3 responses may depend on EPA/DHA ratio, baseline omega‐3 status, supplementation duration, and exercise modality (Fernández‐Lázaro et al. 2024; Heileson et al. 2024; Jäger et al. 2025; Xin and Eshaghi 2021). These sources of heterogeneity limit the development of universal recommendations and instead support individualized, sport‐specific application.

A central interpretive issue highlighted by this review is the recovery–adaptation paradox. Although inflammation and oxidative stress are frequently framed as negative consequences of high‐intensity exercise, these physiological responses also contribute to mitochondrial biogenesis, antioxidant defense adaptation, cellular signaling, and tissue remodeling (Morrison et al. 2015; Paulsen et al. 2014; Peternelj and Coombes 2011; Rogers et al. 2023). Consequently, reductions in inflammatory or oxidative‐stress biomarkers should not automatically be interpreted as superior training outcomes. This issue is particularly relevant in relation to chronic high‐dose antioxidant supplementation. Although vitamins C and E may reduce selected oxidative‐stress markers, evidence indicates that chronic supplementation may blunt important cellular adaptations to endurance training (Morrison et al. 2015; Paulsen et al. 2014; Peternelj and Coombes 2011). Accordingly, caution regarding impaired training adaptation is currently supported primarily by evidence from chronic high‐dose vitamin C and E supplementation rather than by comparable evidence for most functional foods or bioactive compounds. Although modulation of redox or inflammatory signaling by other recovery‐oriented interventions could theoretically influence adaptation, this possibility remains insufficiently established and warrants prospective investigation.

The practical implication of these findings is that recovery nutrition should be periodized. During congested competition schedules, tournaments, travel periods, training camps, or repeated high‐intensity sessions, more aggressive recovery‐oriented nutritional support may be justified because short‐term readiness and performance restoration are prioritized (Kerksick et al. 2017; Naderi et al. 2025; Ranchordas et al. 2017). More aggressive recovery‐oriented nutritional support—defined as the strategic use of evidence‐supported nutritional interventions (e.g., optimized carbohydrate and protein intake together with selected functional foods or bioactive compounds when appropriate) to maximize short‐term recovery—may be justified because immediate readiness and performance restoration are prioritized. The selection of these strategies should also consider athlete characteristics, supplement quality, safety, contamination risk, and compliance with anti‐doping regulations. In contrast, during adaptation‐focused training phases, the objective should not be the complete suppression of inflammatory or oxidative processes, but rather the optimization of recovery while preserving exercise‐induced adaptive signaling (Morrison et al. 2015; Paulsen et al. 2014; Peternelj and Coombes 2011; Rogers et al. 2023). This periodized interpretation represents the principal conceptual contribution of the present review and may assist athletes, coaches, and sport nutrition practitioners in selecting recovery strategies according to physiological demand, recovery window, and performance objectives. To facilitate translation into applied sport settings, Table 5 summarizes the principal functional foods and bioactive compounds, their proposed mechanisms, relevant recovery outcomes, and evidence interpretation.

**TABLE 5:** Functional foods and bioactive compounds for recovery following high‐intensity exercise.

Intervention	Main bioactive component	Proposed mechanism	Most relevant recovery outcomes	Evidence interpretation	Population/context	Typical dose and duration
Carbohydrate	Glucose polymers, sugars, starch	Glycogen resynthesis	Muscle glycogen restoration; subsequent high‐intensity performance	Strong foundational evidence	Exercising individuals/athletes, general	~1.0–1.2 g/kg/h early postexercise when recovery < 4 h
Protein/whey/amino acids	Essential amino acids, leucine	Muscle protein synthesis; tissue repair	Muscle repair; strength recovery; soreness	Strong foundational evidence	Exercising individuals/athletes	1.4–2.0 g/kg/day, ~20–40 g/serving
Tart cherry	Anthocyanins, phenolic acids	Anti‐inflammatory and antioxidant effects	MVC recovery; CRP; soreness; CK	Moderate evidence; effects vary by outcome and protocol	Healthy adults/athletes, strenuous/damaging exercise	1–2 servings/day, 7–16 days (juice or concentrate)
Beetroot	Nitrate, betalains	Nitric oxide availability; blood flow; redox support	Soreness; CMJ; repeated‐sprint recovery	Low‐to‐moderate evidence; context‐specific	Active adults, eccentric/repeated‐sprint exercise	Dietary nitrate via juice/concentrate, acute or short‐term
Pomegranate	Ellagitannins, punicalagins, anthocyanins	Antioxidant and anti‐inflammatory effects	LDH; oxidative stress; soreness	Promising but inconsistent evidence	Healthy/active adults	Juice/extract; dose and duration heterogeneous
Cocoa flavanols	Epicatechin, catechins	Vascular and antioxidant effects	Oxidative‐stress markers; vascular responses	Limited evidence for direct performance recovery	Exercising adults	Flavanol dose, product form, and duration heterogeneous
Green tea	Catechins, EGCG	Antioxidant and anti‐inflammatory effects	TAC; oxidative‐stress markers; fatigue‐related muscle damage	Low‐to‐moderate evidence for redox support	Exercising adults	~400–800 mg/day catechins, > 1 week
Curcumin	Curcuminoids	NF‐κB modulation; anti‐inflammatory and antioxidant effects	DOMS; CK; inflammatory markers	Moderate but formulation‐sensitive evidence	Physically active/athletic populations	~150–1500 mg/day, pre‐ to 72 h postexercise; formulation‐dependent
Cinnamon	Cinnamaldehyde, cinnamic acid, polyphenols	Putative antioxidant, anti‐inflammatory, and metabolic effects	Soreness; oxidative stress; inflammatory markers	Very low evidence in exercise recovery	Iranian female athletes (limited populations studied)	Dose/duration not consistently reported
Omega‐3 fatty acids	EPA, DHA	Pro‐resolving lipid mediators; membrane effects	CK; LDH; myoglobin; soreness; inflammation	Moderate but heterogeneous evidence	Healthy/active adults	~0.5–4 g/day EPA/DHA, 1 to several weeks
Vitamins C and E	Ascorbate, tocopherols	Radical scavenging; redox modulation	Oxidative‐stress markers; cellular adaptation	Cautionary evidence; possible adaptation‐blunting with chronic high‐dose use	Recreationally trained/healthy adults, endurance/strength training	1000 mg/day vitamin C + 235 mg/day vitamin E, 10–11 weeks

Abbreviations: CK, creatine kinase; CMJ, countermovement jump; CRP, C‐reactive protein; DHA, docosahexaenoic acid; DOMS, delayed‐onset muscle soreness; EGCG, epigallocatechin gallate; EPA, eicosapentaenoic acid; LDH, lactate dehydrogenase; MVC, maximal voluntary contraction; NF‐κB, nuclear factor‐kappa B; TAC, total antioxidant capacity.

## 5 Practical Implications

Recovery nutrition should begin with foundational macronutrient adequacy. Carbohydrate intake should be prioritized when rapid glycogen restoration is needed, particularly during same‐day repeated sessions or congested competition schedules. Protein intake should be distributed across the day to support muscle protein synthesis and repair after resistance, sprint, eccentric, or muscle‐damaging exercise. Functional foods and bioactive compounds, including tart cherry, beetroot, curcumin, and omega‐3 fatty acids, may be useful as adjunct strategies during periods of high soreness, repeated competition, or short recovery windows. However, their use should consider dose, timing, formulation, sport context, athlete tolerance, and contamination risk. Antioxidant and anti‐inflammatory strategies should be periodized: they may be useful when short‐term readiness is the priority, but chronic high‐dose antioxidant supplementation should be used cautiously during adaptation‐focused training blocks. The proposed framework should be regarded as a conceptual model derived from narrative evidence synthesis rather than a validated decision algorithm. Future prospective and comparative studies are required to evaluate its applicability across different exercise modalities, athlete populations, and competition settings.

## 6 Limitations

This review has several limitations. First, although a structured search strategy was applied, the review was not registered and did not follow a full systematic review protocol. Second, no formal meta‐analysis was conducted because of heterogeneity in exercise protocols, supplementation regimens, recovery outcomes, biomarker timing, and participant training status. Third, the interpretation of evidence may be influenced by variability in bioactive compound formulation, dose, duration, and timing. Fourth, some included interventions, such as curcumin, omega‐3 fatty acids, and high‐dose antioxidant vitamins, may function more accurately as bioactive supplements than strict functional foods; therefore, the title and interpretation were broadened to include both functional foods and bioactive compounds. Fifth, because this manuscript was designed as a structured narrative review rather than a systematic or scoping review, database yield, duplicate removal, full‐text exclusion counts, and a PRISMA flow diagram were not reported. Finally, the hierarchical and periodized framework proposed in this review has not been prospectively validated in intervention studies. It should therefore be regarded as an author‐derived conceptual synthesis developed from the integration of the reviewed evidence, intended to guide hypothesis‐driven research and evidence‐informed practice rather than to serve as a validated decision model. Future systematic or scoping reviews should include a fully reproducible screening flow, formal risk‐of‐bias assessment, and protocol registration when appropriate.

## 7 Research Gaps and Future Directions

Several important gaps limit the translation of current evidence into applied sport settings. First, many studies still use small sample sizes and recreationally active participants, which limits generalizability to elite and highly trained athletes. Future research should involve larger athlete‐specific samples and consider differences across endurance, intermittent team‐sport, racket‐sport, combat‐sport, and strength‐power athletes. Second, female athletes remain underrepresented in recovery nutrition research. This is a critical limitation because sex‐related differences in substrate metabolism, inflammation, hormonal regulation, muscle damage responses, and supplement metabolism may influence recovery outcomes. Future studies should include sufficient female‐athlete samples and report sex‐specific analyses when possible.

Third, intervention protocols remain highly heterogeneous. Studies vary widely in dose, duration, timing, formulation, and verified bioactive content, particularly for tart cherry, curcumin, beetroot, pomegranate, cocoa flavanols, and green tea. Future trials should standardize or clearly report bioactive compound content, supplementation timing, product formulation, and adherence to improve comparability across studies.

Fourth, exercise models differ substantially across studies. Eccentric resistance exercise, repeated sprinting, HIIT, downhill running, marathon running, and simulated sport activity produce different recovery demands. Therefore, future research should align nutritional interventions with the dominant physiological stressor, such as glycogen depletion, muscle damage, soreness, inflammation, oxidative stress, vascular function, or neuromuscular fatigue. Fifth, biomarker timing remains inconsistent. Markers such as creatine kinase, C‐reactive protein, interleukin‐6, malondialdehyde, and soreness may peak at different time points after exercise. Studies using only one postexercise time point may miss important recovery responses. Future studies should include repeated biomarker assessments across acute and delayed recovery windows, such as immediately postexercise, 24, 48, and 72 h.

Sixth, many studies overemphasize biochemical markers without integrating meaningful performance outcomes. Future research should combine creatine kinase, lactate dehydrogenase, C‐reactive protein, interleukin‐6, tumor necrosis factor‐alpha, malondialdehyde, superoxide dismutase, glutathione peroxidase, and total antioxidant capacity with perceptual and functional measures, including delayed‐onset muscle soreness, maximal voluntary contraction, countermovement jump, repeated‐sprint ability, sport‐specific skill performance, and next‐day training quality. Seventh, cinnamon remains insufficiently studied as an exercise‐recovery bioactive compound. Although its antioxidant, anti‐inflammatory, and metabolic properties are biologically plausible, current exercise‐specific evidence is limited. Future randomized, placebo‐controlled trials should use standardized cinnamon type, dose, duration, and recovery outcomes before cinnamon can be recommended for sport recovery practice.

Future research should prioritize adequately powered randomized controlled trials that directly compare commonly used functional foods under standardized experimental conditions. Greater consistency in exercise protocols, participant characteristics, supplementation dose, timing, duration, and formulation would improve the comparability of findings across studies. In addition, future investigations should evaluate clinically relevant recovery outcomes, including subsequent exercise performance, neuromuscular function, return‐to‐training readiness, and athlete‐reported recovery, alongside established biochemical markers of inflammation and oxidative stress. Such studies would help identify the contexts in which specific functional foods provide meaningful recovery benefits and support the development of evidence‐based, sport‐specific nutritional recommendations. Finally, future research should examine Indonesian and broader Asian athlete populations. Dietary patterns, body composition, supplement access, culinary habits, and exposure to locally available functional foods may differ from Western athlete samples. This is particularly relevant for turmeric, cinnamon, ginger, tropical fruits, fermented foods, and other regionally available bioactive food sources.

## 8 Conclusions

Based on the integrated evidence synthesized in this review, we propose a hierarchical and periodized conceptual framework for recovery nutrition following high‐intensity exercise. This framework represents an author‐derived synthesis intended to support evidence‐informed nutritional decision‐making rather than a validated decision model. Carbohydrate and protein remain the most robust and transferable strategies because they directly support glycogen restoration and skeletal muscle repair. Functional foods and bioactive compounds should be viewed as adjuncts rather than replacements for foundational sports nutrition practices. Among the reviewed interventions, tart cherry, curcumin, omega‐3 fatty acids, and beetroot show the most practical potential for selected recovery outcomes, although their effects vary according to dose, timing, formulation, exercise model, and outcome measure. Pomegranate, cocoa flavanols, and green tea appear more consistent for oxidative‐stress modulation than for direct performance recovery, whereas cinnamon remains an emerging compound with insufficient exercise‐recovery evidence. Chronic high‐dose vitamin C and E supplementation should be approached cautiously because potential reductions in oxidative stress may be accompanied by blunted training adaptations.

The most defensible approach is to secure carbohydrate and protein adequacy first, then apply functional foods and bioactive compounds selectively according to the athlete's recovery window, dominant physiological stressor, competition schedule, and adaptation goal. Future research should use standardized dosing, verified bioactive content, athlete‐specific samples, repeated biomarker assessments, and functional performance outcomes to clarify how functional foods and bioactive compounds can be integrated into applied recovery nutrition. Future prospective intervention studies should evaluate whether this conceptual framework improves recovery outcomes compared with current nutritional practice across different exercise modalities, athlete populations, and competitive settings.

## Author Contributions

**Haydar Rafsya Farzana:** conceptualization, writing – review and editing, writing – original draft, methodology, software, formal analysis, resources, data curation, investigation. **Gosy Endra Vigriawan:** supervision, investigation, writing – review and editing, software, data curation. **Antonello Santini:** conceptualization, investigation, writing – original draft, writing – review and editing, supervision. **Fahrul Nurkolis:** conceptualization, investigation, writing – original draft, writing – review and editing, visualization, validation, methodology, software, formal analysis, supervision.

## Funding

The authors have nothing to report.

## Consent

The authors have nothing to report.

## Conflicts of Interest

The authors declare no conflicts of interest.

## Data Availability

Data sharing is not applicable to this article as no datasets were generated or analyzed during this study.
